# Food container employing a cold atmospheric plasma source for prolonged preservation of plant and animal origin food products

**DOI:** 10.1016/j.mex.2020.101177

**Published:** 2020-12-08

**Authors:** Panagiotis Dimitrakellis, Marianna Giannoglou, Angelos Zeniou, Evangelos Gogolides, George Katsaros

**Affiliations:** a Institute of Nanoscience and Nanotechnology, NCSR “Demokritos”, Aghia Paraskevi 15341, Attiki, Greece; b Institute of Technology of Agricultural Products, Hellenic Agricultural Organization-DEMETER, Lykovrissi 14123, Attica, Greece

**Keywords:** Cold atmospheric plasma, Food container, Surface dielectric barrier discharge, Decontamination, Shelf-life extension, Food sanitization

## Abstract

Cold Atmospheric Plasma is a non-thermal processing technology with great potential for application to food products as it can effectively reduce the microbial load, leading to substantial shelf-life extension. Herein, we present an easy-to-build and cost-effective Surface Dielectric Barrier Discharge (SDBD) plasma source adjusted to the plastic lid of a common commercial food container made of transparent glass. Implementation and evaluation of plasma treatment in real perishable food products such as sea bream fillets, fresh-cut rocket salads and fresh whole strawberries showed that such device might be efficiently used in-storage for the extension of their shelf-life.

• Easy-to-build and cost-effective SDBD plasma source adjusted in a food container for generation of antimicrobial RONS in proximity to treated food product
• Treatment of perishable food products by reducing their initial microbial load
• In-storage treatment efficient for perishable food products shelf-life extension

## Graphical abstract

**Figure:**
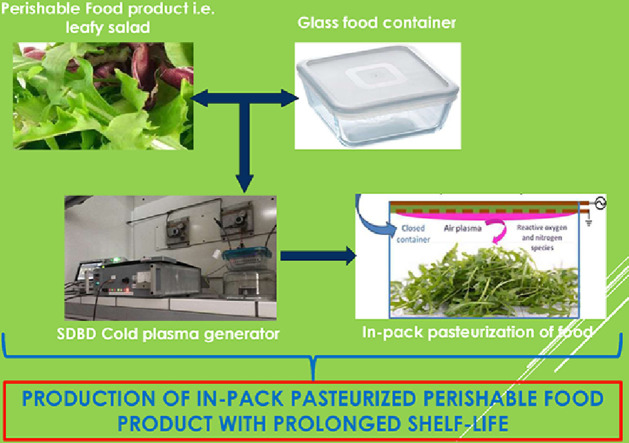

 Specifications table

**Table:** 

Subject Area:	Agricultural and Biological Sciences
More specific subject area:	Food science and technology
Method name:	Plasma device for in-storage food preservation
Name and reference of original method:	N/A
Resource availability:	N/A

**Method details**

## Background

Plant and animal origin foods such as fruits, vegetables and fresh fish, as well as their products are highly perishable due to their high initial microbial load and improper handling and storage conditions; hence the shelf-life extension with minimal food waste along the whole value chain is of primary importance for sustainable food production and distribution. As a result, efficient and non-destructive processing of all these products is highly desired for prolonging their shelf-life, while minimally affecting their nutritional and quality characteristics.

Non-thermal processing technologies could be utilized either as thermal treatment substitutes or where conventional pasteurization is not applicable (i.e. to fresh fish fillets). Among these techniques, Cold Atmospheric Plasma (CAP) in air-rich environment is particularly interesting due to its efficiency to inactivate microorganisms in low temperature without significantly affecting the food quality indices [1]. CAPs are generated by electrical discharges using several different plasma sources such as Dielectric Barrier Discharges (DBDs) and Plasma Jets [2]. Air plasmas produce several reactive oxygen and nitrogen species (RONS), such as O_3_, O_2_^−^, OH, NO and H_2_O_2_, that are well known for their broad antimicrobial activity against bacteria and fungi [3,4].

Of particular interest for food decontamination purposes are the Surface Dielectric Barrier Discharges (SDBD), a special configuration of DBD plasma sources where the two metallic electrodes are adjusted to the opposite sides of a single dielectric material [5,6]. CAP is sustained only at the electrode-air interface and typically appears in the form of arrays of several individual sub-millimeter discharges, often called surface micro-discharges (SMDs) [7,8]. SDBDs in ambient air can be applied for remote food treatment where the food is not in direct contact with the active plasma region. In such case the food is not affected by UV radiation and charged particles and only the neutral species are participating in the decontamination process, ensuring ‘mild’ processing conditions with less heating and minimal effect on quality indices and nutritional attributes. From the engineering point of view, SDBDs are very promising devices due to the construction simplicity, the plasma generation in ambient air at relatively low voltage values and with low cost equipment, the uniform distribution of reactive neutral species over the whole material surface and the up-scaling potential [5,6]. On the other hand, it is the mass transport of the long-lived reactive species, mainly O_3_, N_2_O_5_, HNO_3_ and N_2_O, that determines the decontamination efficiency [9], [10], [11]. In order to enhance mass transport of reactive species, even the short-lived ones, high gas flows are employed vertically to the target to enhance convection of species in the downstream region and different electrode geometries are studied to optimize directional mass transport [6,8].

## Food box plasma device fabrication

**Fig. 1:**
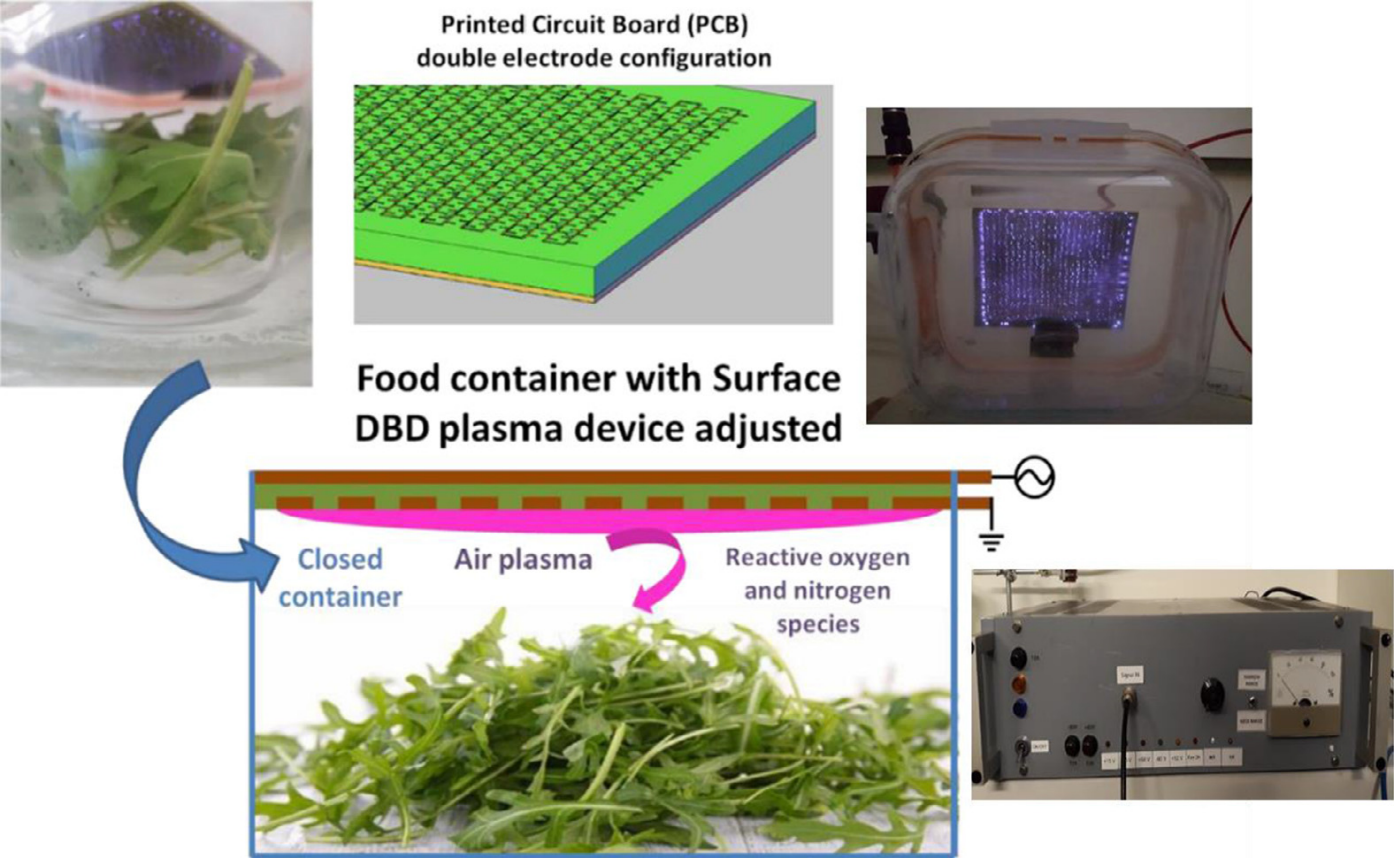
Configuration of the commercial food container made of transparent glass with a tight and leak proof plastic lid, which accommodates a Surface Dielectric Barrier Discharge (DBD) source.

Our approach entails the in-storage food decontamination and shelf-life extension using a food container that employs a Cold Atmospheric Plasma (CAP) source. Such device provides efficient generation of antimicrobial RONS, while confining them inside the food container and in proximity to the treated food product. We used a commercial, square-shaped food container (1-L volume), comprising a transparent glass container and a tight and leak-proof plastic lid. A large-area, 12 × 12 cm, rectangular Surface Dielectric Barrier Discharge (SDBD) source was adjusted on the inside surface of the lid. The SDBD source is based on a patented stacked electrode configuration on a Printed Circuit Board (PCB) [12]. The PCB electrode has a 40 µm thick copper plane in one side, serving as the powered electrode that is connected to a high voltage power supply. The copper plane is in contact with a dielectric layer of 1 mm thickness, made of glass fiber reinforced epoxy composite material. This material serves as the dielectric barrier between the HV copper electrode and the grounded grid electrode. The latter is a 40 µm thick copper grid, carefully machined in order to maximize electric field [13]. This patented electrode design has already been applied in radio-frequency driven volume DBD device for polymer surface treatments [14,15]. It is the first time that this electrode is used to create surface discharges using low frequency AC high voltages. The surface discharge was generated in ambient air confined inside the food box using AC high voltage at 6 KV peak-to-peak and frequency 45 kHz. The sinusoidal voltage signal was created using a signal generator and amplified using sequentially a power amplifier and a high voltage transformer. The HV plane electrode is glued to the inner surface of the lid, while the grounded grid electrode is facing the inner volume where the food is placed. Both electrodes are connected to the external cables through electrical connections made by drilling the plastic lid. The schematics of the PCB electrode as well as the whole food container configuration during treatment of leafy products are presented in Fig. 1.

## Characterization

**Fig. 2:**
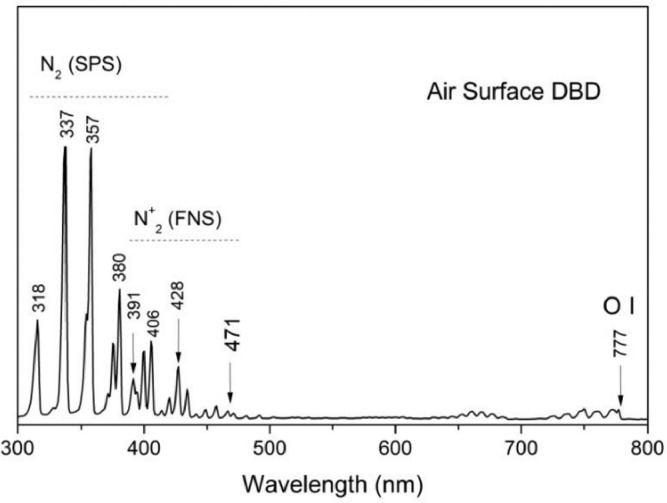
Optical emission spectrum of the air plasma on the surface of the PCB-based plasma source that is adjusted inside the commercial food container.

For the gas phase characterization of air plasma, optical emission spectroscopy (OES) measurements were performed using an Ocean Optics 65000 spectrophotometer and a Vis-near-infrared optical fiber. The emission spectra were recorded using the Spectra Suite software of Ocean Optics and the recorded data were corrected according to the spectral efficiency of the optical fiber (Fig. 2). The spectrum is dominated by the peaks related to the N_2_ Second Positive System (SPS) and N_2_^+^ First Negative System, mostly due to the very high concentration of molecular nitrogen in air atmosphere. On the contrary, the emission from the atomic oxygen lines is almost negligible due to the rapid depopulation of excited atomic oxygen in air plasmas. The chemistry of such discharges is typically dominated by ozone (O_3_) and several long-lived NO_x_ species that contribute synergistically to the decontamination process at the downstream region. OES measurements cannot directly identify the formation of ozone, while the NO peaks only appear in the UV region (out of spectral range of the current study). However, the very intense bands of N_2_ SPS in the 318 - 380 region are indicative of the highly populated excited levels nitrogen molecules; the excitation of molecular nitrogen to vibrationally excited levels, with cross sections way higher than the direct dissociation to atomic nitrogen, is considered the most efficient pathway toward nitrogen fixation to NO_x_ species in air or oxygen-rich plasmas. Therefore, similar to all surface barrier discharges in air environment, the formation of nitrogen oxides is highly anticipated [6,16].

## Method validation

Fish, fruits and vegetables were treated inside the container and the CAP effect on microbial load and quality indices was evaluated upon storage. Samples of fish fillets, leafy salads and fresh whole strawberries were used as food model products. Treatment of 10 min for sea bream fillets and 15 min for rocket salad and fresh whole strawberries was applied using the CAP food container. The aforementioned food samples were then removed from the container and stored at refrigeration conditions (2–6 °C) along with untreated samples. Total microbial load and quality characteristics were analyzed exactly after treatment and during storage at various time intervals, allowing for the shelf-life estimation of all treated and untreated samples.

Perishable food products were selected for the evaluation of the efficacy of CAP treatment in the food container. Sea bream fillets [17], fresh-cut rocket leaves [18] and whole fresh strawberries were the products tested under the CAP inside the food container. Total plate count load was the main quality index for the comparison of CAP treated and untreated samples immediately after treatment. For the microbiological analysis, 10 g from each sample were diluted in 90 mL of sterile Ringer solution and homogenized for 1 min in sterile plastic bags. TVC was enumerated on PCA (pour plate method, 3 days incubation at 25 °C). In Table 1, the total microbial load of the aforementioned food products before and after CAP treatment is presented. CAP treatment resulted in significant initial load decrease for all samples tested, ranging from 16% for the fresh-cut rocket leaves to 36% for whole fresh strawberries. This decrease in the total microflora is expected to have effect on the shelf-life of the products.

**Table 1:** Total microbial load of untreated and Cold Atmospheric Plasma (CAP) treated samples of fish fillets, fresh-cut rocket leaves and whole fresh strawberries along with the estimated corresponding percentage of initial load reduction (means± standard deviation).

Food product	Total microbial load (logCFU/g)		Initial microbial load reduction (%)
	Untreated	CAP	
Sea Bream fillets	5.57 ± 0.07	4.54 ±0.04	18.5
Rocket leaves	6.38 ± 0.11	5.36 ± 0.13	16.0
Strawberries	2.34 ± 0.10	1.45 ± 0.21	38.0

Comparative shelf-life tests (among CAP treated and untreated samples) were performed, measuring all major quality deterioration factors for each product. Based on dominant deterioration factor for each product, the shelf-life was estimated. For sea bream fillets and fresh-cut rocket leaves, the total microflora was the dominant factor, while for strawberries, the corresponding factor was organoleptic deterioration combined with total phenolic content. In Table 2 the shelf-life for each sample is presented after storage at specific temperatures.

**Table 2:** Shelf-life estimation of untreated and Cold Atmospheric Plasma (CAP) treated samples of fish fillets, fresh-cut rocket leaves and whole fresh strawberries after storage at specific temperatures.

Food Product	Storage Temperature (°C)	Estimated Shelf-life (h)		Shelf-life Extension (%)
		Untreated	CAP	
Sea Bream fillets	4	120	180	50
Rocket leaves	2	63	116	84
Strawberries	6	235	326	39

For the fish fillets, a 50% shelf-life increase was observed, while for the fresh-cut rocket leaves the corresponding percentage was higher than 80% and for the whole fresh strawberries approximately 40%. In general, the CAP treated samples were superior compared to untreated ones, based on main quality indices for each product.

## Conclusion

In conclusion, the proposed concept of a food container employing a cold atmospheric plasma source can be used for the treatment of perishable food products, aiming to reduce the initial microbial load and consequently to extend the shelf-life. Furthermore, the container used (or similar containers) may allow for even more shelf-life extension if the lid is not opened after treatment (for food products that oxygen is not a prerequisite for their storage stability).

## Declaration of Competing Interest

The authors declare that they have no known competing financial interests or personal relationships that could have appeared to influence the work reported in this paper.

## References

[1] Niemira B.A. (2012). Cold plasma decontamination of foods. Ann. Rev. Food Sci. Technol..

[2] Pappas D. (2011). Status and potential of atmospheric plasma processing of materials. J. Vac. Sci. Technol..

[3] Laroussi M. (2005). Low temperature plasma‐based sterilization: overview and state‐of‐the‐art. Plasma Processes Polym..

[4] Lu X., Naidis G.V., Laroussi M., Reuter S., Graves D.B., Ostrikov K. (2016). Reactive species in non-equilibrium atmospheric-pressure plasmas: generation, transport, and biological effects. Phys. Rep..

[5] Dickenson A., Morabit Y., Hasan M.I., Walsh J.L. (2017). Directional mass transport in an atmospheric pressure surface barrier discharge. Sci. Rep..

[6] Hasan M.I., Walsh J.L. (2017). Influence of gas flow velocity on the transport of chemical species in an atmospheric pressure air plasma discharge. Appl. Phys. Lett.

[7] Shimizu T., Sakiyama Y., Graves D.B., Zimmermann J.L., Morfill G.E. (2012). The dynamics of ozone generation and mode transition in air surface micro-discharge plasma at atmospheric pressure. New J. Phys..

[8] Luan P., Oehrlein G.S. (2018). Stages of polymer transformation during remote plasma oxidation (RPO) at atmospheric pressure. Appl. Phys..

[9] Sakiyama Y., Graves D.B., Chang H.W., Smimitzu T., Morfill G.E. (2012). Plasma chemistry model of surface microdischarge in humid air and dynamics of reactive neutral species. Appl. Physics.

[10] Dickenson A., Britun N., Nikiforov A., Leys C., Hasan M.I., Walsh J.L. (2018). The generation and transport of reactive nitrogen species from a low temperature atmospheric pressure air plasma source. PCCP.

[11] Bartis E.A.J., Knoll A.J., Luan P., Seog J., Oehrlein G.S. (2016). On the interaction of cold atmospheric pressure plasma with surfaces of bio-molecules and model polymers. Plasma Chem. Plasma Process..

[12] Gogolides E., Zeniou A., Dimitrakellis P., Large-area, uniform, atmospheric pressure plasma processing device. EPO application No 16386016.6 (2017), EP 3-142-467-A1.

[13] Dimitrakellis P., Zeniou A., Stratakos Y., Gogolides E. (2016). Radio frequency atmospheric plasma source on a printed circuit board for large area, uniform processing of polymeric materials. Plasma Sources Sci. Technol..

[14] Dimitrakellis P., Gogolides E., Zeniou A., Awsiuk K., Rysz J., Marzec M.M. (2017). Transition between stable hydrophilization and fast etching/hydrophilization of poly(methyl)methacrylate polymer using a novel atmospheric pressure dielectric barrier discharge source. J. Vac. Sci. Technol..

[15] Dimitrakellis P., Patsidis A.C., Smyrnakis A., Psarras G.C., Gogolides E. (2019). Atmospheric plasma nanotexturing of organic–inorganic nanocomposite coatings for multifunctional surface fabrication. ACS Appl. Nano Mater..

[16] Walsh J.L., Liu D.X., Iza F., Rong M.Z., Kong M.G. (2010). Contrasting characteristics of sub-microsecond pulsed atmospheric air and atmospheric pressure helium–oxygen glow discharges. J. Phys. D: Appl. Phys..

[17] Giannoglou M., Dimitrakellis P., Efthimiadou A., Gogolides E., Katsaros G. (2020). Comparative study on the effect of cold atmospheric plasma, ozonation, pulsed electromagnetic fields and high pressure technologies on sea-bream fillets quality indices and shelf-life. Food Eng. Rev..

[18] Giannoglou M., Stergiou P., Dimitrakellis P., Gogolides E., Stoforos N., Katsaros G. (2020). Effect of cold atmospheric plasma processing on quality and shelf life of ready-to-eat rocket leafy salads. Innovative Food Sci. Emerg. Technol..

